# Real-life relevant face perception is not captured by the N170 but reflected in later potentials: A comparison of 2D and virtual reality stimuli

**DOI:** 10.3389/fpsyg.2023.1050892

**Published:** 2023-03-28

**Authors:** Merle Sagehorn, Marike Johnsdorf, Joanna Kisker, Sophia Sylvester, Thomas Gruber, Benjamin Schöne

**Affiliations:** ^1^Experimental Psychology I, Institute of Psychology, Osnabrück University, Osnabrück, Germany; ^2^Semantic Information Systems Research Group, Institute of Computer Science, Osnabrück University, Osnabrück, Germany

**Keywords:** face perception, N170, virtual reality, late potentials, realistic conditions, ecological validity, EEG

## Abstract

The perception of faces is one of the most specialized visual processes in the human brain and has been investigated by means of the early event-related potential component N170. However, face perception has mostly been studied in the conventional laboratory, i.e., monitor setups, offering rather distal presentation of faces as planar 2D-images. Increasing spatial proximity through Virtual Reality (VR) allows to present 3D, real-life-sized persons at personal distance to participants, thus creating a feeling of social involvement and adding a self-relevant value to the presented faces. The present study compared the perception of persons under conventional laboratory conditions (PC) with realistic conditions in VR. Paralleling standard designs, pictures of unknown persons and standard control images were presented in a PC- and a VR-modality. To investigate how the mechanisms of face perception differ under realistic conditions from those under conventional laboratory conditions, the typical face-specific N170 and subsequent components were analyzed in both modalities. Consistent with previous laboratory research, the N170 lost discriminatory power when translated to realistic conditions, as it only discriminated faces and controls under laboratory conditions. Most interestingly, analysis of the later component [230–420 ms] revealed more differentiated face-specific processing in VR, as indicated by distinctive, stimulus-specific topographies. Complemented by source analysis, the results on later latencies show that face-specific neural mechanisms are applied only under realistic conditions (A video abstract is available in the Supplementary material and via YouTube: https://youtu.be/TF8wiPUrpSY).

## 1. Introduction

As an inherently social species, humans highly rely on their ability to appraise faces. The human brain is specialized in recognizing and interpreting faces, as cortical regions, like, e.g., the fusiform face area and cells in the inferior temporal cortex, are especially sensitive to face stimuli (Mccarthy et al., 1997; Nestor et al., 2008; Pyles et al., 2013; Weiner and Zilles, 2016; Palejwala et al., 2021), enabling the extraction of social information as well as the reaction to, and the interaction with the social environment. The cognitive and emotional mechanisms underlying these abilities have evolutionary evolved in a socially complex and responsive environment. Specifically, they have been attuned to social situations in which at least two individuals are present and engaging in face-to-face communication. A person directly addressing another communicates information and usually expects a verbal or non-verbal response (Keltner and Kring, 1998; Keltner et al., 2003). Thus, the physical proximity preconditioning social involvement facilitates the facial information to immediately become self-relevant. When being actively involved in a social situation, it is imperative to extract affective information from facial expressions to deduce the other persons’ emotions, intentions and expectations (Keltner and Kring, 1998; Hamm et al., 2003; Matsumoto et al., 2008). A situation in which a person has to process facial expressions conveying emotions and intentions that do not hold any relevance, virtually do not occur in real-life and thus do not correspond to the normal operating mode of neuronal face recognition mechanisms. In a real-life situation, for example, it is impossible for anger, reflected by facial expressions, to not possess interpersonal meaning for the recipient.

However, this rather observational approach, where participants are confronted with faces that do not bear any or only little meaning, constitutes the conventional laboratory paradigm employing 2D-monitor presentation of faces. To put it differently, in the conventional laboratory participants observe faces, but even though these faces look directly at them, the participants are not seen by anyone. Under real-life conditions, this is a highly improbable scenario. For the sake of experiential control, facial stimuli are even further reduced to their basic physical attributes. Conventional laboratory experiments study face perception by means of gray scale pictures usually eliminating head shape and hair style (e.g., Jemel et al., 2003; Miyoshi et al., 2004; Blau et al., 2007; Dering et al., 2011), and investigate effects of inversion, i.e., faces presented upside down (e.g., Rossion et al., 1999; Itier and Taylor, 2004; Vizioli et al., 2010). Paying tribute to the complexity of real-life face perception, more ecological valid approaches use colored pictures of whole scenes (Rousselet et al., 2004), dynamic faces and face animations (Recio et al., 2011) or videos featuring faces (Johnston et al., 2015). Still, typical face stimuli are not only limited in realism concerning properties such as resolution, color, perspective and size. They are also not part of an egocentric reference frame – only the laboratory setup they are being presented in is – and as aforementioned devoid from any social context. Therefore, face perception might only be examined as an isolated process. Given the apparent discrepancies between real-life and laboratory setups, the neural mechanisms observed in a 2D environment might be domain specific and not exhibit the same functional properties in a realistic setting.

Virtual Reality (VR) is technically capable of increasing the realism of classical laboratory designs by allowing for presentation of stimuli in real-world size, offering depth structure and spatial proximity (Parsons, 2015; Pan and Hamilton, 2018; Snow and Culham, 2021; Schöne et al., 2021b; Kisker et al., 2021b). Previous studies employing VR as a method for direct comparison of cognitive and emotional mechanisms under conventional laboratory as opposed to realistic conditions found significant deviations between the two modalities. Long-standing effects established by various classical laboratory experiments could not be replicated in VR, respectively (Schöne et al., 2021a,b; Kisker et al., 2021c). Specifically, replicating Simons and Chabris (1999) seminal invisible gorilla paradigm in VR revealed that inattentional blindness plays a much more subordinate role than the original experiment might have implicated (Schöne et al., 2021b). Not only attentional processes change their operational mode under realistic conditions, but also memory encoding and retrieval work differently. The well-established theta old/new-effect only occurs when remembering pictural stimuli, whereas recognition of scenes where the participant was actually present relies on different mnemomic processes (Kisker et al., 2021b). Also, a comparison of emotional and motivational markers by means of frontal alpha asymmetries provides evidence that the models derived from laboratory data cannot be applied to realistic settings without restrictions (Schöne et al., 2021a; Kisker et al., 2021c). These differences between laboratory and VR settings can be ascribed to the fact that VR is technically capable to create a highly realistic and thus self-relevant experiences (e.g., Gabana et al., 2018; Schöne et al., 2019, 2021a; Kisker et al., 2021b; Newman et al., 2022). Perceived realism in virtual environments can be derived from observing behavior during virtual experiences (e.g., Blascovich et al., 2002; Gromer et al., 2018; Xu et al., 2021; Kisker et al., 2021a,c) as well as neural responses (Schöne et al., 2023). Moreover, the autobiographic mnemonic mechanisms guiding retrieval as their employment suggest that those experiences are remembered as if they were real (Schöne et al., 2019, 2021a; Kisker et al., 2021c).

So far, only few studies have investigated face perception using VR, focusing on context modulations (Stolz et al., 2019) and emotional valence encoding in face perception (Kirasirova et al., 2021). Up to our knowledge, however, none have included the comparison between laboratory and realistic conditions. Thus, as a first step towards a more holistic understanding of face perception in an ecological valid setting, we translated the classical laboratory setup into a VR setting, enhancing realism with the goal of maintaining strict experimental control (Parsons, 2015; Snow and Culham, 2021). To this end, we presented images of people on a 2D monitor to the participants in a blocked within-design, and the very same scene as an immersive 3D virtual experience in which the presented people sat directly in front of the participants.

To ensure maximal comparability of the results obtained under VR and 2D conditions and to be able to integrate them into the vast body of electrophysiological scientific literature on face perception, we followed the overall rationality of laboratory conventions, i.e., sequential randomized presentation of stimuli in a controlled environment. Although the sequential presentation of up-popping static persons in the physical vicinity of the participants is physically still highly improbable, this setup bridges the gap between the conventional laboratory and a more realistic approach to face perception tackling the issue of neglecting involvement and self-relevance.

Most importantly, it allows for comparing the canonical event-related potentials (ERPs) associated with face perception. The most discussed neural correlate of perceptual processing of human faces is the ERP component N170, which has been related to face perception and categorization based on observed stimulus-dependent amplitude differences (Rossion and Jacques, 2011). It is characterized by a negative deflection in amplitude occurring at about 170 ms after presentation of a face that can be measured at occipito-temporal electrode positions, i.e., over posterior visual cortical areas (Eimer, 2011; Rossion and Jacques, 2011). Stronger, i.e., more negative, N170 amplitudes have been shown to occur for faces compared to objects and for emotional faces compared to neutral faces (e.g., Itier and Taylor, 2004; Blau et al., 2007). Still, further results showed that the N170 is also sensitive to other objects (e.g., cars; Dering et al., 2009; Boehm et al., 2011), dependent on expertise (e.g., Bukach et al., 2006; Ip et al., 2017), also influenced by perceptual variety of the stimulus material (e.g., Thierry et al., 2007; Dering et al., 2009; Boehm et al., 2011) and not able to differentiate human and ape faces (Zion-Golumbic and Bentin, 2007). Source localization of the N170 points to the fusiform gyrus (Mccarthy et al., 1997; Herrmann et al., 2005b; Rossion and Jacques, 2011) and the superior temporal sulcus region (STS; Itier and Taylor, 2004) while brain activation patterns additionally involve sources in a parieto-temporal-occipital network (Herrmann et al., 2005b).

Another component potentially relevant in face perception is the P1, an early positive component occurring at around 100 ms post-stimulus at occipito-temporal electrodes. Albeit the P1 has been shown to mostly be sensitive to various low-level perceptual properties of visual stimuli (e.g., stimulus contrast), direction of spatial attention and arousal state (Rossion et al., 1999; Jemel et al., 2003), it has also been associated with face perception, as some studies report a categorial sensitivity towards faces (Itier and Taylor, 2002; Herrmann et al., 2005a; Thierry et al., 2007; Dering et al., 2009; Kuefner et al., 2010).

Modulation by social relevance (Bublatzky et al., 2014), contextual and self-related emotion (Herbert et al., 2013; Stolz et al., 2019) as well as decision-relevant information (Ratcliff et al., 2009), can only be observed in later components (> 250 ms). While the N170 component is clearly involved in basic face categorization to some degree, more profound processing resulting in a global, individualized and highly informative face representation is manifested in other electrophysiological correlates than just one early ERP component (Zion-Golumbic and Bentin, 2007). The vast majority of scientific studies nevertheless focus on the N170 and fewer studies report results on later components related to face perception along with early potentials. However, these studies indicate that profound processing of faces beyond basic sensory-perceptual properties is rather captured by late potentials (Ratcliff et al., 2009; Herbert et al., 2013; Bublatzky et al., 2014; Stolz et al., 2019). Consequently, the process of perceiving and interpreting a face under realistic conditions is not necessarily limited to the early components.

Due to the above-mentioned increase in spatial proximity, realism and thus self-relevance of persons when presented in VR, we expected more sophisticated and in-depth face processing under realistic conditions. This could already be evident in better discrimination between faces and controls under realistic conditions, as reflected by increased amplitude differences between stimulus types for the early components, particularly the N170. However, given the previously reported doubts about the face specificity of the N170, we rather expected less discrimination between faces and controls under realistic conditions, reflected in absence of early amplitude differences between stimulus types in VR.

We instead hypothesized, based on the aforementioned results on later components in face processing, that a more realistic encounter with a person should lead to enhanced sensitivity processing of facial features, i.e., better discrimination between faces and controls, in VR, especially reflected by later components (Ratcliff et al., 2009; Herbert et al., 2013; Bublatzky et al., 2014; Schindler et al., 2017; Stolz et al., 2019).

Complementing the ERP analyses, we investigated the neural generators of realistic face perception and expected a larger network of neural structures involved in the underlying processes than under conventional conditions (e.g., Vuilleumier and Pourtois, 2007). We hypothesized that the fusiform gyrus, which is specialized in face perception and object recognition (Mccarthy et al., 1997; Weiner and Zilles, 2016; Palejwala et al., 2021), and the inferior temporal gyrus, associated with higher visual processing and face individuation (Nestor et al., 2008; Pyles et al., 2013) would be part of the network. In addition, we expected to obtain sources related to higher cognitive functions indicating more profound face processing with regard to self-referential and emotional information under realistic conditions.

Since there are no results yet on a direct comparison of face perception under 2D and realistic conditions, our hypotheses concerning specific amplitude and topographical differences between the two modalities remain of overall exploratory nature.

## 2. Methods

### 2.1. Participants

Thirty participants were recruited from Osnabrück University. All participants were screened for psychological and neurological disorders and regular drug use. Only participants who met the inclusion criteria were eligible for the experiment. Additionally, previous experience with computer games and Virtual Reality and the recent usage of such media was documented. Participants had none to little experience with VR and did not wear a VR headset regularly within the last 4 weeks. If vision correction was necessary, only participants wearing contact lenses, not glasses, could participate. It was furthermore ensured that participants had not also been photographed for the stimulus creation (see 2.2) or knew any of the people whose pictures were presented to them (max. Recognition rate was below 7%, i.e., eight out of 120 faces). All participants gave informed written consent. Participants received either partial course credits or 15€ for their participation.

Four participants had to be excluded from participation and analyses due to unmet anamnesis criteria (*n* = 2) or because they aborted the experiment (*n* = 2). Ultimately, 26 data sets were selected for data analyses (*M*_age_ = 22.96 years, SD_age_ = 3.1 years, 20 female, 25 right-handed). The sample size is similar to other studies that conducted a VR experiment featuring face stimuli and also investigated the N170 as well as later components (Stolz et al., 2019).

### 2.2. Stimulus material

**Figure 1:**
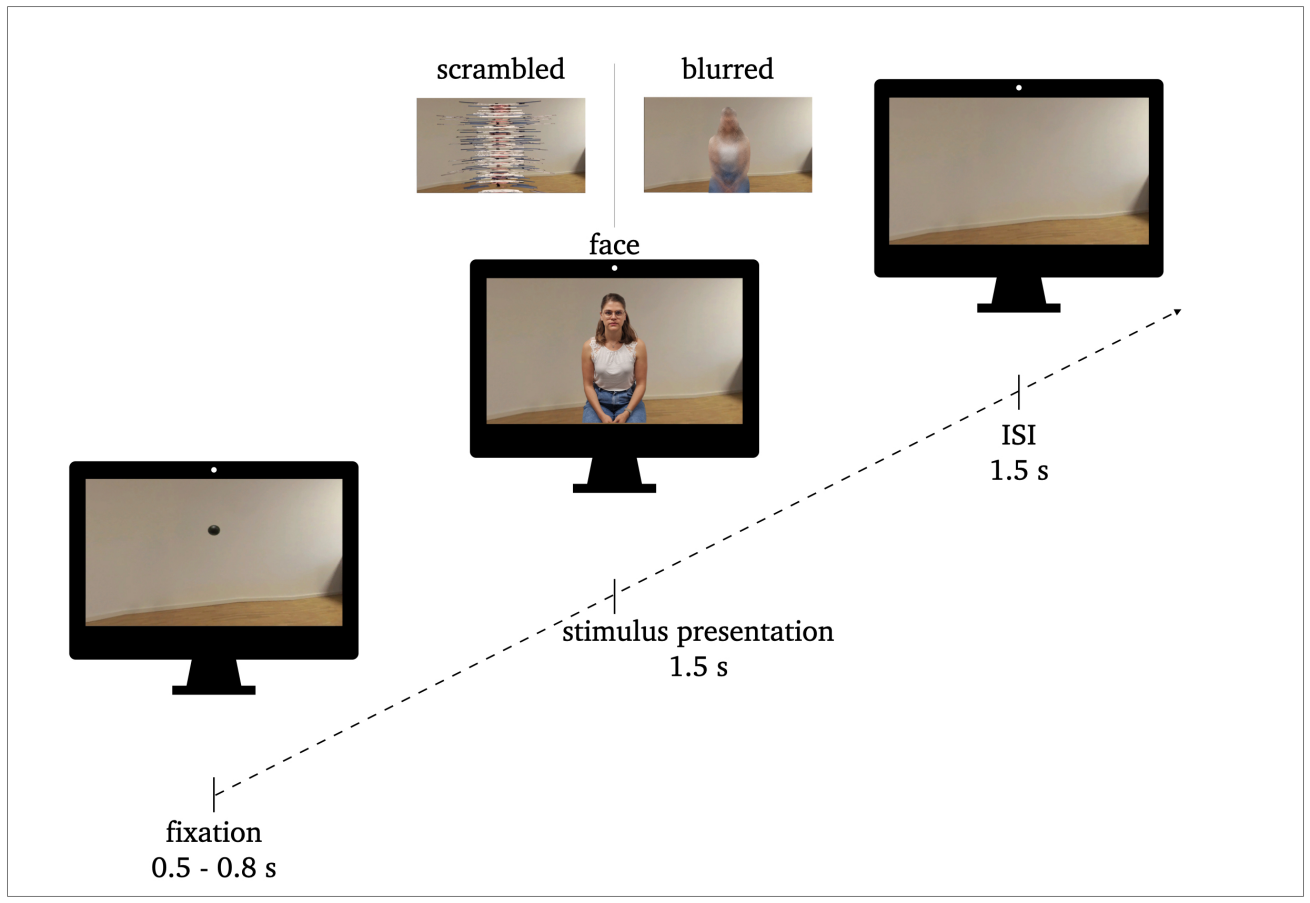
Procedure of stimulus presentation: 0.5–0.8 s fixation, 1.5 s stimulus presentation, 1.5 s inter stimulus interval (ISI). Exemplary stimuli of face conditions and perceptual control conditions (scrambled, blurred) are illustrated.

The stimulus material comprised 120 pictures of persons sitting on a stool in a plain living room as the background, and were rendered as both, 2D and 3D-360° images. All of them showed neutral facial expressions. All images were recorded with the Insta360Pro VR-camera with 8 k resolution at a distance of 62 cm to the person being photographed. The images were randomly assigned per participant to the two conditions (PC or VR), yielding in 60 individual images per condition, while ensuring that participants would not see the same person twice. In addition, two classical perceptual control pictures were presented, which are generally utilized in face processing research in order to control for the perceptual features of the stimuli (e.g., Herrmann et al., 2005a,b; Schwaninger et al., 2009; Kuefner et al., 2010; Bombari et al., 2013; Rossion, 2014; Civile et al., 2018). The first control was a blurred version conserving color information and perceptual frame, i.e., stimulus size and shape, but without the same semantic relevance. The blurring was achieved by applying the glass-filter in Adobe Photoshop 2022 (Distortion filtering: Glass Filter, Distortion: 15, Smoothing: 1, Structure: Milk Glass, Scaling 200%). The second control was a scrambled version sustaining equivalent low-level perceptual visual properties (see Figure 1). For the scrambled pictures, the original pictures were cut in to stripes with a height of 10 pixels that were randomly rearranged in the vertical dimension.

The subtraction of the process of perceiving the blurred or scrambled image from the process of perceiving the normal person isolates the processes actually associated with person perception that are not influenced by the physical attributes of the stimuli like, e.g., size, color or spatial configuration. Controlling for these perceptual factors, i.e., having a perceptual baseline, thus allows for direct comparison of the processes across the different domains (PC and VR), so that the face specific processing reflected by ERP amplitude differences can be compared across modalities.

Taken together, each condition included 180 stimuli (60 normal, 60 blurred, 60 scrambled). The normal persons and their matching control images were always presented in the same modality, respectively.

### 2.3. Procedure

All participants completed both, the PC and the VR condition, while the order of both conditions was alternated between participants. Both conditions were conducted in the same soundproof and electrically shielded room suitable for Electroencephalographic (EEG) measurements. Thus, participants did not switch their location between conditions and were given a five-minute break to relax and get ready for the second condition. During this break, the EEG signal quality was checked. For both conditions, participants were instructed to passively watch the stimulus presentation and keep their movement to a minimum.

For the PC condition, participants were seated in front of a standard PC monitor (24″, 1920 × 1200 resolution) with a constant distance of 115 cm to the screen, resulting in a horizontal viewing angle of 5° and a vertical viewing angle of 2.5°. The pictures were presented in 2D in the center of the screen with a size of 10 × 15 cm using Matlab for stimulus presentation.

For the VR condition, participants remained seated and were equipped with a VR headset (HTC Vive Pro 2, 2448 × 2448 pixel per eye, up to 120° field of vision, 120 Hz refresh rate). The pictures were presented in 3D-360° in real-life size at a distance of 62 cm (horizontal viewing angle: 98°; vertical viewing angle: 42°) *via* the video-game engine Unity 5 (Version 2020). Triggers were sent by Unity and synchronized using Lab Streaming Layer for Unity (LSL by SCCN).^2^

Each of the 180 trials of each condition followed the same sequencing (see Figure 1). The pictures were presented for 1.5 s. They were preceded by a fixation dot (0.5–0.8 s) and followed by an interstimulus interval (ISI; background image without person; 1.5 s). The participants were instructed to blink or move only during the ISI, i.e., while the room they saw was empty. Each trial lasted between 3.5 to 3.8 s resulting in a total run time of approximately eleven minutes per condition. The sequence of the stimulus presentation was the same for both conditions, the only difference was the modality (PC vs. VR). In VR, the participants remained in the same environment (i.e., the living room) for the entire time. The fixation dot and the stimuli appeared in front of them in the respective sequence without any changes to their surroundings. For the ISI the room stayed empty.

Due to the sensitivity of EEG data to motion-induced artifacts, participants were asked to keep motion to a minimum and refrain from looking around in the VR environment. The feasibility of EEG measurements while additionally wearing a head-mounted display (HDM) has been investigated before with special concern for EEG signal quality. However, EEG signal quality was not influenced negatively and the combination of VR using HDM and EEG was rated feasible (Hertweck et al., 2019; Tauscher et al., 2019).

### 2.4. Electrophysiological recordings and preprocessing

An electroencephalogram (EEG) with 128 electrodes, attached in accordance with the international 10-20-system was recorded for the duration of the whole experimental procedure (PC and VR condition). The Active-Two amplifier system from BioSemi (Amsterdam, Netherlands) was used. The sampling rate was 512 Hz, the bandwidth (3 dB) 104 Hz. Additionally, horizontal electrooculogram (hEOG) and vertical electrooculogram (vEOG) were recorded and a common mode sense (CMS) and a driven right leg (DRL) electrode were applied. During the PC condition, the EEG was recorded on the investigators’ computer using ActiView702 Lores. For the VR condition, the trigger stream from Unity was transmitted to Lab Streaming Layer to synchronize the EEG data stream and Unity triggers.

The first preprocessing step necessary only for the VR-condition comprised the merging of the EEG data stream and trigger stream *via* the EEGLAB add-on MoBi-Lab (Ojeda et al., 2014). All further preprocessing steps were applied to the recordings of both modalities using EEGLAB (Delorme and Makeig, 2004).

The data were re-referenced to average reference, high-pass filtered at 0.25 Hz and low-pass filtered at 30 Hz. Bad channels were identified using the automatic channel removal add-on (ASR; Mullen et al., 2015) and interpolated. All channels were linearly detrended for elimination of extended potential drifts. Artifact rejection was applied using independent component analysis (ICA; Delorme et al., 2007). Specifically, an automatic automated ICA component labeling was performed (ICLabel v1.4; Pion-Tonachini et al., 2019; artifact selection confidence of 90% for Muscle, Heart, Line Noise und Channel Noise and 80% for Eye Artifacts). The results of the ICA were visually verified. For epoching, the time window around the trigger onset was set from −500 to 1,500 ms and the baseline correction was set from −300 to 0 ms before trigger onset. Per modality and within modality per stimulus category (face, blurred, scrambled), grand means were computed resulting in six individual ERPs (i.e., VR-face, VR-blurred, VR-scrambled, PC-face, PC-blurred, PC- scrambled).

### 2.5. ERP components in electrode space

**Figure 2:**
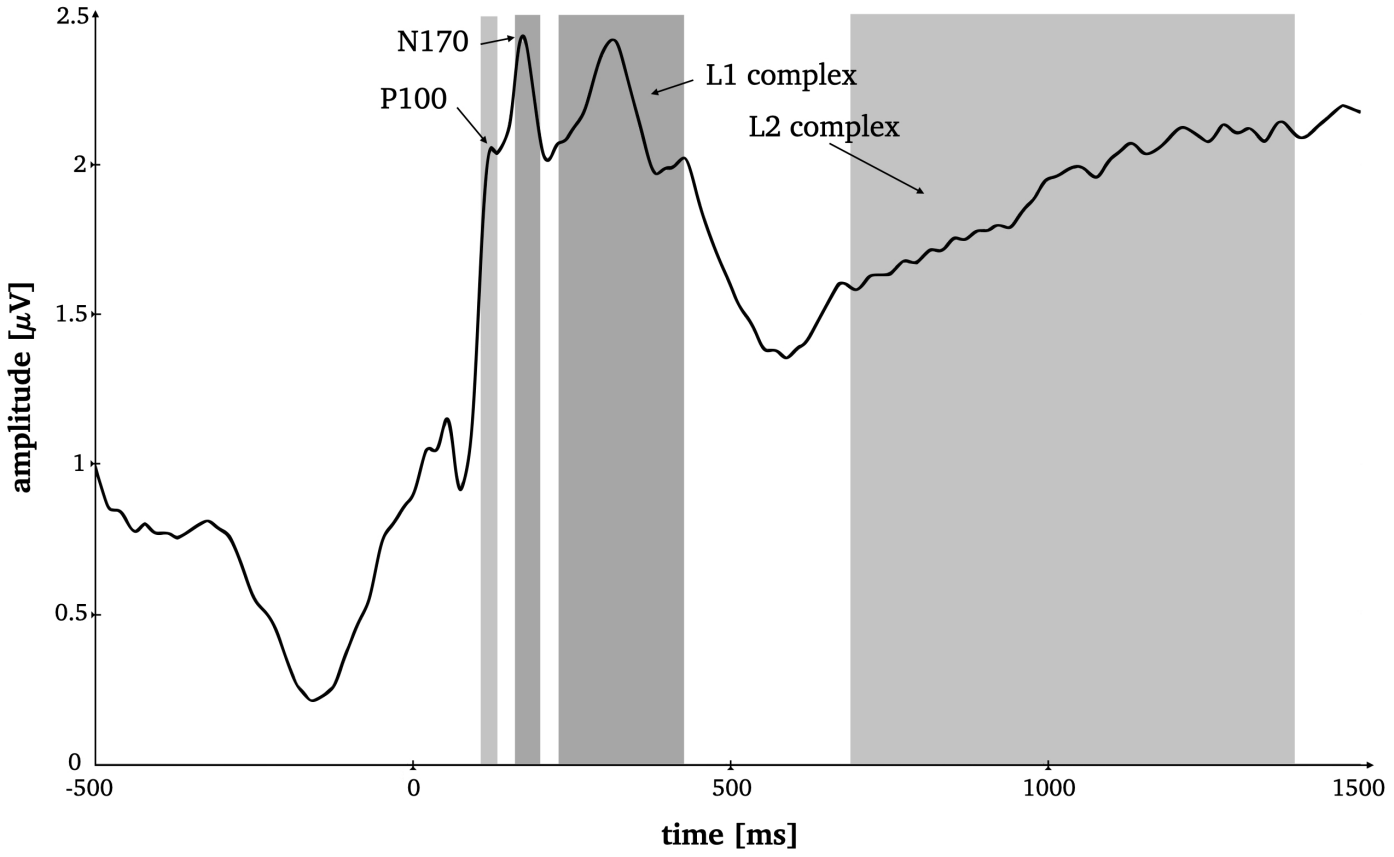
Time-by-amplitude plot of the root mean squared ERP averaged over all electrodes for the selection of appropriate time windows for all ERP components. Grey highlighted sections mark the time windows for P1 (95–125 ms), N170 (165–195 ms), L1 (230–420 ms) and L2 (685–1,385 ms).

**Figure 3:**
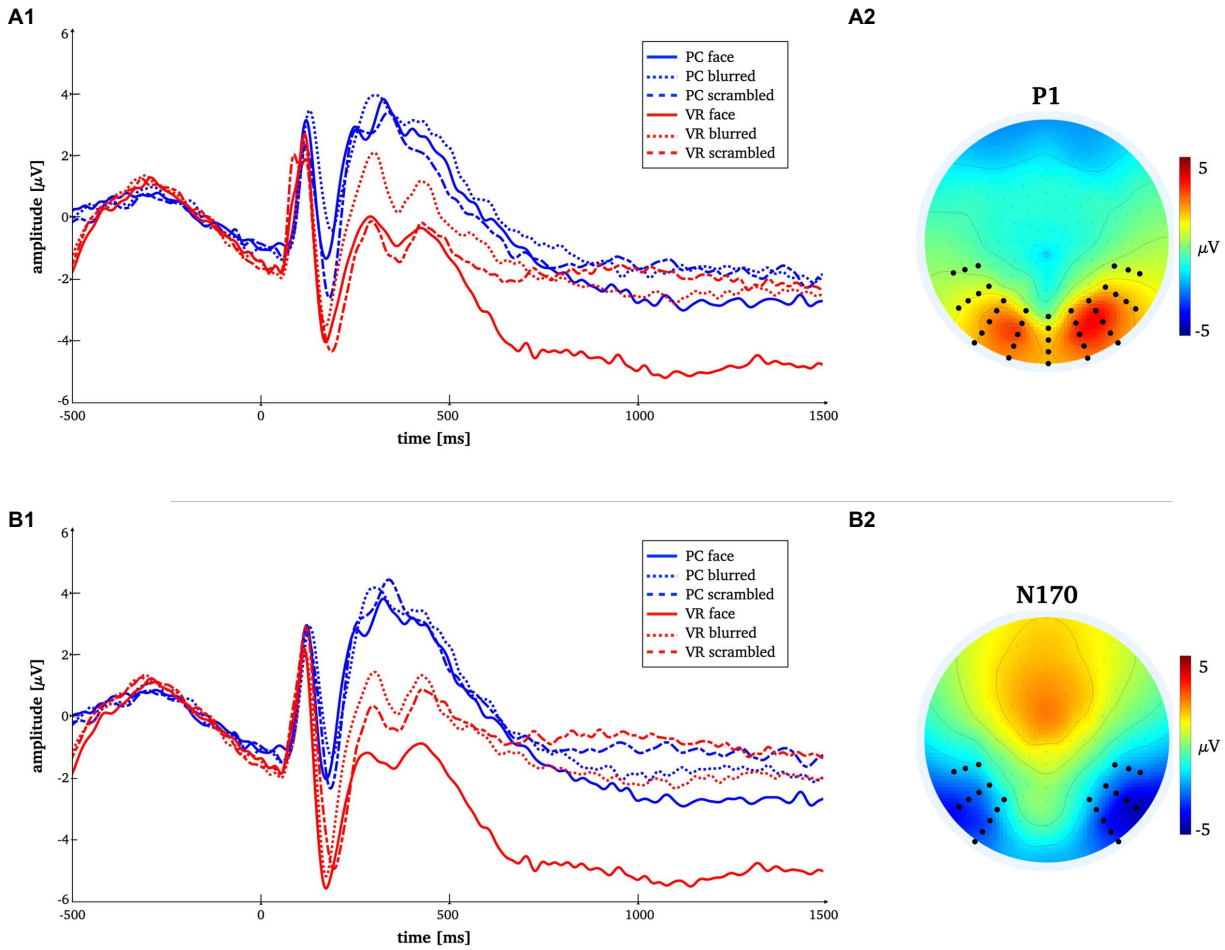
Time-by-amplitude plot of the mean P1 and N170 amplitudes for all conditions [panels **A1,B1**]. Mean topographies across conditions used for ERP averaging [panel **A2,B2**]. The electrodes selected for analyses are indicated. For the P1 electrodes Oz, O1, O2, P7, PO7, P8, PO8, TP7, TP8 and those in close vicinity were used. For the N170 electrodes P7, P8, PO7, PO8, P10, P9, PO10, PO9, TP7, TP8 and those in close vicinity were used.

The time windows and electrode sites for the classical ERP components were selected based on prior literature (Latinus and Taylor, 2006; Rossion and Jacques, 2008; Boehm et al., 2011; Dering et al., 2011), as well as visual inspection of the root-mean-squared ERP (see Figure 2) and the mean topographies across modality and stimulus type (see Figure 3) of all conditions. The P1 was analyzed at posterior midline and lateral (i.a., Oz, O1, O2) as well as occipito-parietal (i.a., PO7, PO8, P7, P8) electrodes in a 95–125 ms time window. The N170 was analyzed at parietooccipital electrodes (i.a., P7, P8, P9, P10, PO7, PO8, PO9, PO10). Here, the time window was set from 165 to 195 ms. The P1 and the N170 amplitudes were computed by calculating the mean voltage across the selected electrodes and time windows, respectively.

Two more consecutive potential complexes were selected for analysis, both analyzed at all 128 electrode positions. Prior literature on late potentials in face and object perception served as basis for our hypotheses on time window selection (Bublatzky et al., 2014; Stolz et al., 2019; Johnsdorf et al., 2023), which was ultimately decided upon based visual inspection of the root mean squared ERP (Figure 2). The L1 component was set from 230 to 420 ms around the local maximum approximately corresponding to the known early posterior negativity potential (EPN; Bublatzky et al., 2014). The L2 was set from 685 to 1,385 ms which comprises a late local minimum and the consecutive amplitude increase (see Figure 2) approximately corresponding to the knows late positive potential (LPP; Bublatzky et al., 2014).

### 2.6. Statistical analysis

All data preparation for statistical testing, i.e., generating respective means for selected time windows and electrodes, as well as the correlation analyses was implemented in Matlab (Version R2021b). The rmANOVA was done *via* IBM SPSS Statistics (Version 27), while *post hoc t*-testing was additionally double-checked in Matlab. For additional robust statistical testing the statistic software R (Version 4.2.1) was used.

#### 2.6.1. P1 and N170

The EEG data for P1 and N170 were analyzed using a 2 × 3 repeated-measurements ANOVA (rmANOVA) with the within-subject factors “modality” (VR vs. PC) and “stimulus type” (face vs. blurred vs. scrambled). Whenever necessary, Greenhouse–Geisser-corrected *p*-values are reported. Significant effects of rmANOVA were complemented by *post hoc t*-tests within each modality, as well as for between-modality interactions. For the N170, we complement our analysis with a robust rmANOVA employing the same factors as well as robust *post hoc t*-tests (both without bootstrapping). For the robust rmANOVA, we used the wwtrim function from the WRS package with default parameters, i.e., trim = 0.2, and the yuend function from the WRS2 package for the *t*-tests (Mair and Wilcox, 2020). Robust statistics can be used to ensure replicability with small sample sizes.

#### 2.6.2. L1 and L2

For L1 and L2, the EEG data were analyzed using 2D-correlation (see formula below) as a first step to determine whether similarities between the topographies of the stimulus types were comparable in both conditions:

$$r_{2D}=\frac{\sum_{m}\sum_{n}\left(A_{mn}-\bar{A}\right)\left(B_{mn}-\bar{B}\right)}{\sqrt{(\sum_{m}\sum_{n}\left(A_{mn}-\bar{A}{)}^{2}\right)\left(\sum_{m}\sum_{n}{\left(B_{mn}-\bar{B}\right)}^{2}\right)}}$$

Additionally, Pearson correlation coefficients across all 128 electrodes were calculated per participant for all within-modality comparisons and also for relevant between-modality comparisons. The mean correlation coefficient and the number of significant correlations was determined for all comparisons. To test statistically whether similarities between stimulus types varied between modalities, the within-modality comparisons were then *t*-tested against respective pairings of the other modality (i.e., PC face ~ PC blurred vs. VR face ~ VR blurred).

### 2.7. ERP components in source space

To determine the differences in activation of the cortical generators involved in face perception under conventional laboratory conditions compared to realistic conditions, variable resolution electromagnetic tomography (VARETA; Bosch-Bayard et al., 2001) was applied. VARETA provides an intracranial distribution of current densities in source space that is spatially smoothest and highly compatible with the amplitude distribution in electrode space (Gruber et al., 2006; Martens et al., 2011). We applied an inverse solution that comprised 3,244 grid points arranged in a 3D grid. The grid was defined by a Leadfield matrix and corresponded to the placement of the 128-channel EEG-system (10-20-system).

**Figure 4:**
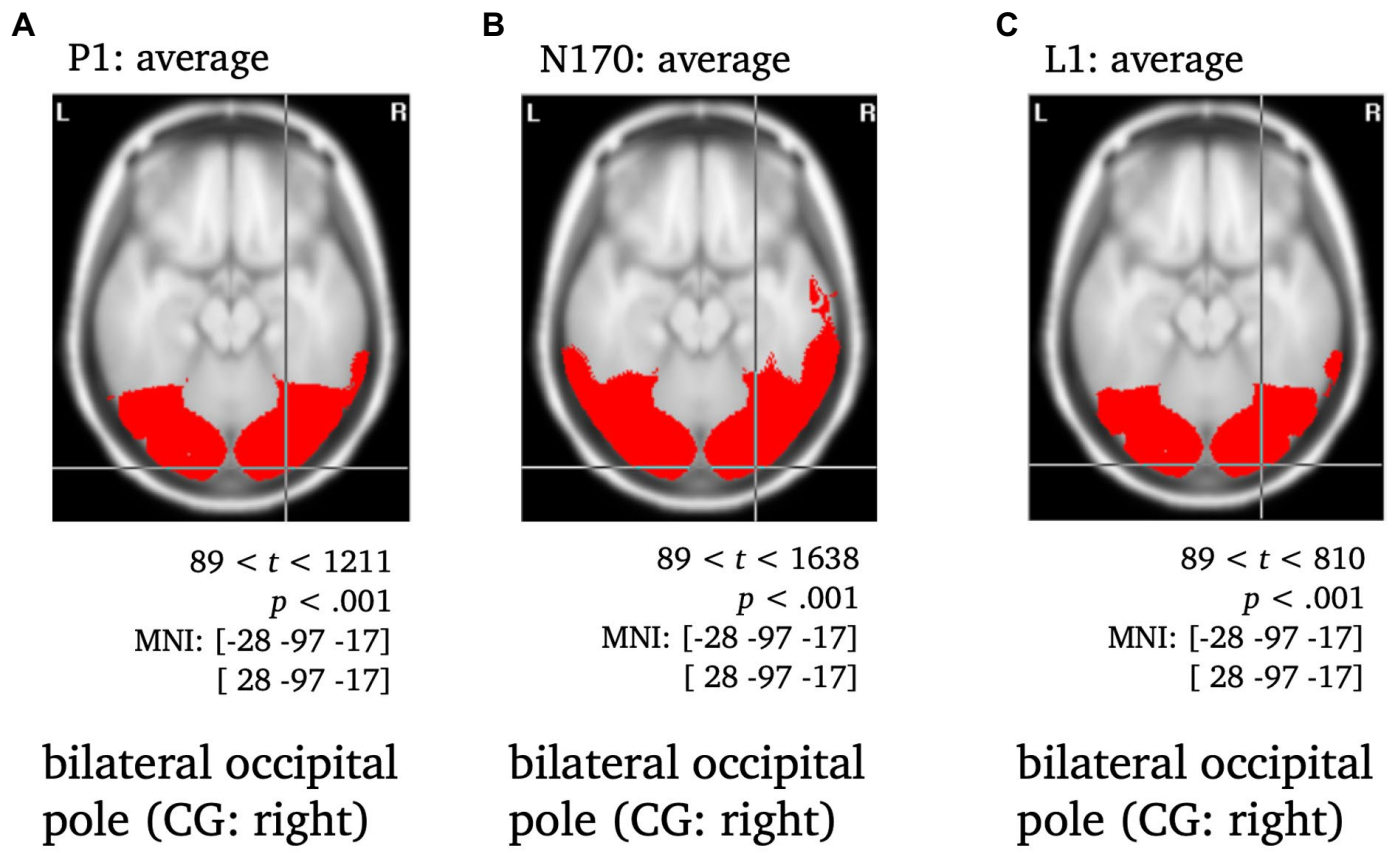
Statistically significant sources for the time span of the P1, N170 and L1 averaged across conditions. Statistically significant differences in activity are marked red, with *p* < 0.001, *t*_crit_ = 89. Per panel, the center of gravity (CG) is labeled and the respective MNI coordinates are given.

**Figure 5:**
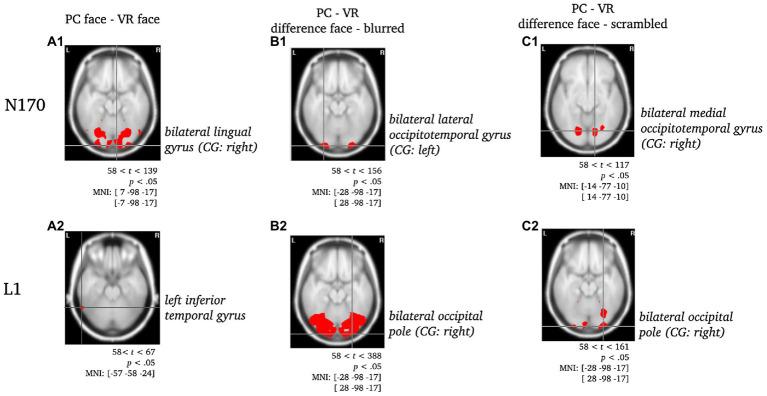
Statistically significant sources for the difference between PC-faces and VR-faces during N170 and L1. Statistically significant differences in activity are marked red, with *p* < 0.05, *t*_crit_ = 58. Per panel, the center of gravity (CG) is labeled and the respective MNI coordinates are given. Per component, the raw comparison of PC faces and VR faces is depicted [panels **A**]. Moreover, this comparison was controlled for blurred faces [panels **B**; PC (face - blurred) *minus* VR (face - blurred)] and scrambled faces [panels **C**, PC (face - scrambled) *minus* VR (face - scrambled); *cf.* ERPs in electrode space].

**Figure 6:**
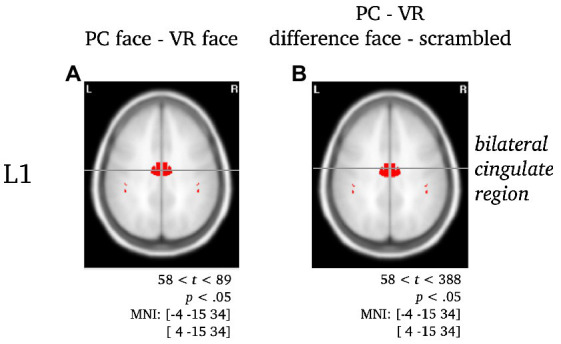
Separate visualization of the cingulate region during L1 for the difference between stimuli presented in PC or VR. Statistically significant differences in activity are marked red, with *p* < 0.05, *t*_crit_ = 58. The center of gravity (CG) is labeled and the respective MNI coordinates are given.

To localize differences in activation patterns, *Hotellings T^2^*-test was performed per effect of interest (see ERP components in electrode space). As a first validation step, the sources of the P1, N170 and L1 components of the ERP were localized to validate the use of VARETA with the current data set (see Figure 4). For all *t*-tests regarding the average across all conditions, the critical *t*-value was *t*_crit_ = 89 with a significance level of *p* < 0.001. After a consistency check against previous publications (e.g., Di Russo et al., 2002; Gruber et al., 2006), the sources of further effects of interest were examined. For these comparisons between conditions (see N170, L1; Figures 5, 6) the significance level was set to *p* < 0.05 and the critical *t*-value was *t*_crit_ = 58. Significant voxels were projected onto the cortical surface which was constructed on the basis of the average probabilistic MRI brain atlas by the Montreal Neurological Institute (MNI; Evans et al., 1993). The brain region’s names for significant voxels were identified by the brain electrical tomography (BET) Neuronic Tomographic viewer.

## 3. Results

### 3.1. ERP components in electrode space

#### 3.1.1. P1

**Figure 7:**
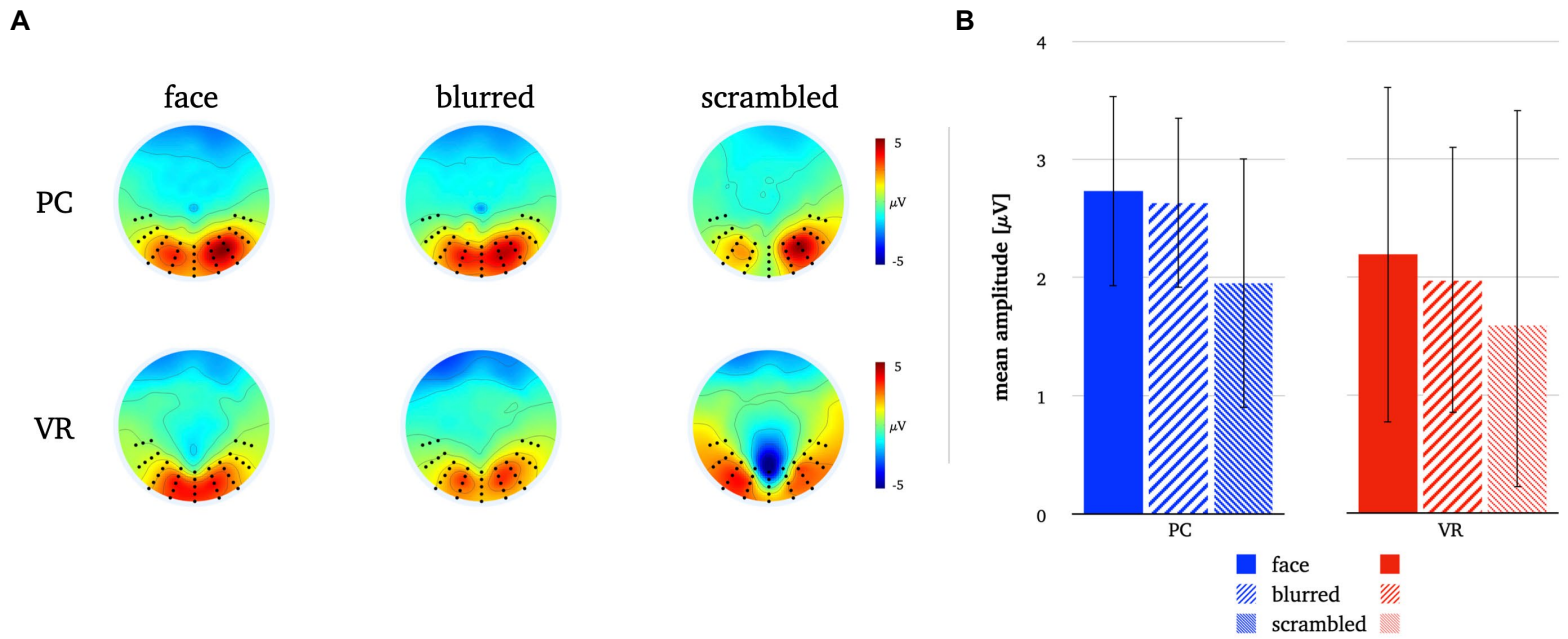
Panel **A** illustrates the P1 topographies for all stimulus types in both modalities. Panel **B** depicts the mean P1 amplitudes for all stimulus types in both modalities. The error bars depict the confidence intervals for the mean values. Significant differences within each modality are marked (*p < 0.05).

The rmANOVA for the P1 component revealed no significant main effects for the factors “modality” and “stimulus type” or the interaction of both (*F*_modality_(1, 25) = 0.84, *p* = 37; *F_stimulus_*(2, 50) = 2.7, *p* = 0.09, ε = 0.78; *F_interaction_*(2, 50) = 0.29, *p* = 0.75). The respective descriptive statistics are given in Table 1 and Figure 7.

**Table 1:** P1 and N170 mean amplitudes, standard deviations and confidence intervals for both modalities and all stimulus types.

	*M*	*SD*	Confidence interval	
			Lower limit	Upper limit
**P1 – PC**				
Face	2.73	2.0	1.93	3.53
Blurred	2.63	1.8	1.92	3.35
Scrambled	1.95	2.6	0.9	3.0
**P1 – VR**				
Face	2.19	3.5	0.77	3.61
Blurred	1.97	2.8	0.85	3.1
Scrambled	1.59	4.5	−0.22	3.41
**N170 – PC**				
Face	−1.48	2.9	−2.63	−0.32
Blurred	−0.87	3.0	−2.1	0.4
Scrambled	−0.183	3.4	−3.2	−0.5
**N170 – VR**				
Face	−5.11	4.0	−6,72	−3.5
Blurred	−4.39	3.8	−5.94	−2.85
Scrambled	−4.28	4.4	−6.05	−2.52

#### 3.1.2. N170

The rmANOVA for the N170 component revealed significant main effects for the factor “modality” (*F_modality_*(1, 25) = 49.27, *p* < 0.001, *η^2^* = 0.66), but not for the factor “stimulus type” (*F*_stimulus_(2, 50) = 1.85, *p* = 0.178, ε = 0.75), while the interaction of “modality” and “stimulus type” was significant (*F_interaction_*(2, 50) = 3.87, *p* = 0.029, *η^2^* = 0.25). The respective descriptive statistics are given in Table 1 and the results of the robust ANOVA are given in Table 2.

**Table 2:** Results of 2 × 3 repeated-measurements ANOVA (rmANOVA) with the within-subject factors “modality” (VR vs. PC) and “stimulus type” (face vs. blurred vs. scrambled).

		*df*	*F*	*p*	partial *η^2^*
Modality	Standard	1	49.27	<0.001***	0.66
	Robust	2	57.79	<0.001***	-
Stimulus type	Standard	2	1.85	0.167	0.07
	Robust	1	3.03	0.05*	-
Interaction	Standard	2	3.87	0.027*	0.13
	Robust	2	0.29	0.075	-

For every comparison, robust *F*-statistics are given in the second line. Significant effects are marked (*p < 0.05; **p < 0.01; ***p < 0.001).

**Figure 8:**
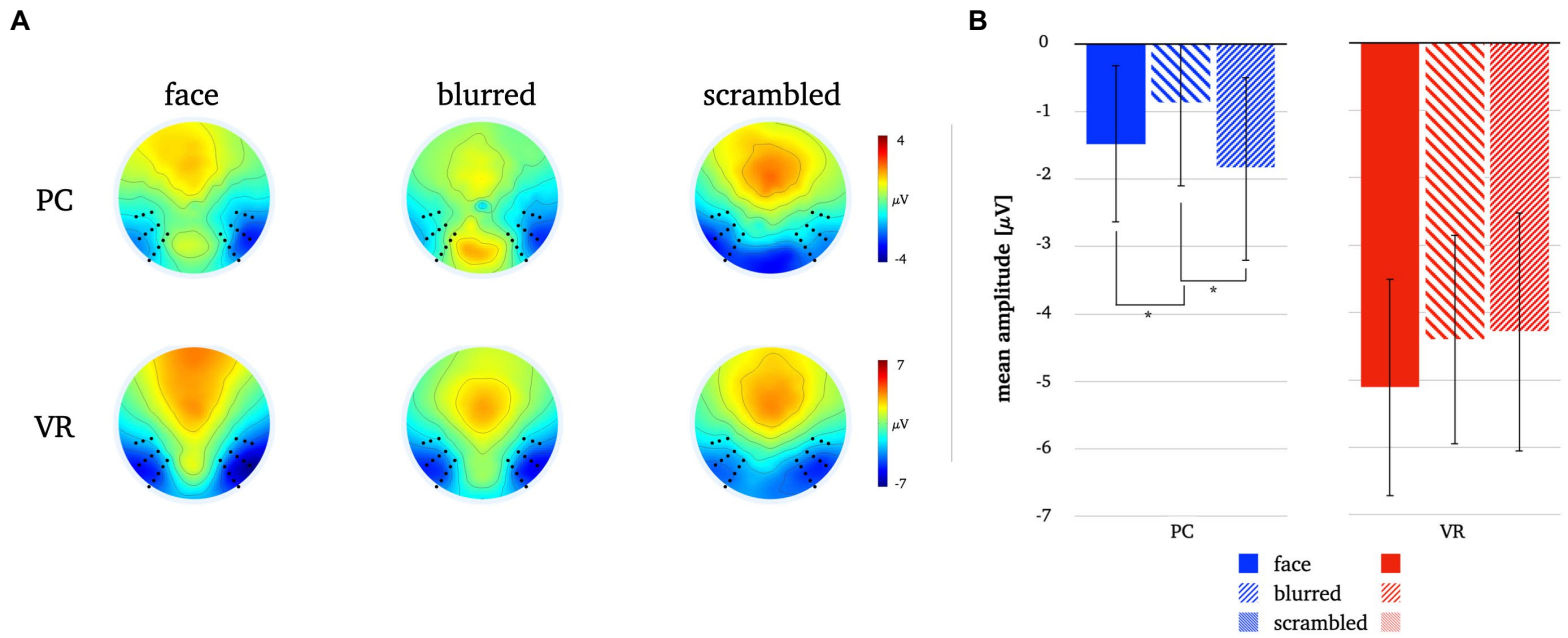
Panel **A** illustrates the N170 topographies for all stimulus types in both modalities. Panel **B** depicts the mean N170 amplitudes for all stimulus types in both modalities. The error bars depict the confidence intervals for the mean values. Significant differences within each modality are marked (*p < 0.05).

A significantly more negative N170 amplitude was found for normal compared to blurred persons [*t*(25) = −2.08, *p* = 0.048, *d* = −0.41] and for scrambled compared to blurred persons [*t*(25) = 2.94, *p* = 0.007, *d* = 0.58] in the PC modality, but no significant difference in amplitude for normal compared to scrambled persons [*t*(25) = −0.08, *p* = 0.434]. In VR, there were no significant N170 amplitude differences between stimulus types (all *ps* > 0.05, see also Figure 8; Table 3).

**Table 3:** Pairwise comparisons of N170 amplitudes within and between modalities.

		*df*	*t*	*p*	Cohen’s *d*
**PC**					
Face – Blurred	Standard	25	−2.08	0.048*	−0.41
	Robust	15	−2.64	0.019*	0.18
Face – Scrambled	Standard	25	0.08	0.434	0.16
	Robust	15	−0.53	0.605	0.06
Blurred – Scrambled	Standard	25	2.94	0.007**	0.58
	Robust	15	1.31	0.21	0.11
**VR**					
Face – Blurred	Standard	25	−1.83	0.08	−0.36
	Robust	15	−1.611	0.128	0.15
Face – Scrambled	Standard	25	−1.48	0.151	−0.29
	Robust	15	−1.01	0.326	0.12
Blurred – Scrambled	Standard	25	−0.24	0.813	−0.05
	Robust	15	0.15	0.885	0.01
**PC versus VR**					
Face	Standard	25	6.17	<0.001***	1.21
	Robust	15	5.22	<0.001***	0.62
Blurred	Standard	25	4.35	<0.001***	1.72
	Robust	15	8.89	<0.001***	0.61
Scrambled	Standard	25	3.65	<0.001***	0.83
	Robust	15	5.25	<0.001***	0.5
Face – Blurred	Standard	25	0.26	0.797	0.05
	Robust	15	−0.32	0.752	0.05
Face – Scrambled	Standard	25	2.37	0.026*	0.47
	Robust	15	1.45	0.167	0.25
Blurred – Scrambled	Standard	25	2.05	0.035*	0.44
	Robust	15	2.03	0.061	0.36

For every comparison, robust *t*-statistics are given in the second line. Significant differences are marked (*p < 0.05; **p < 0.01; ***p < 0.001).

**Figure 9:**
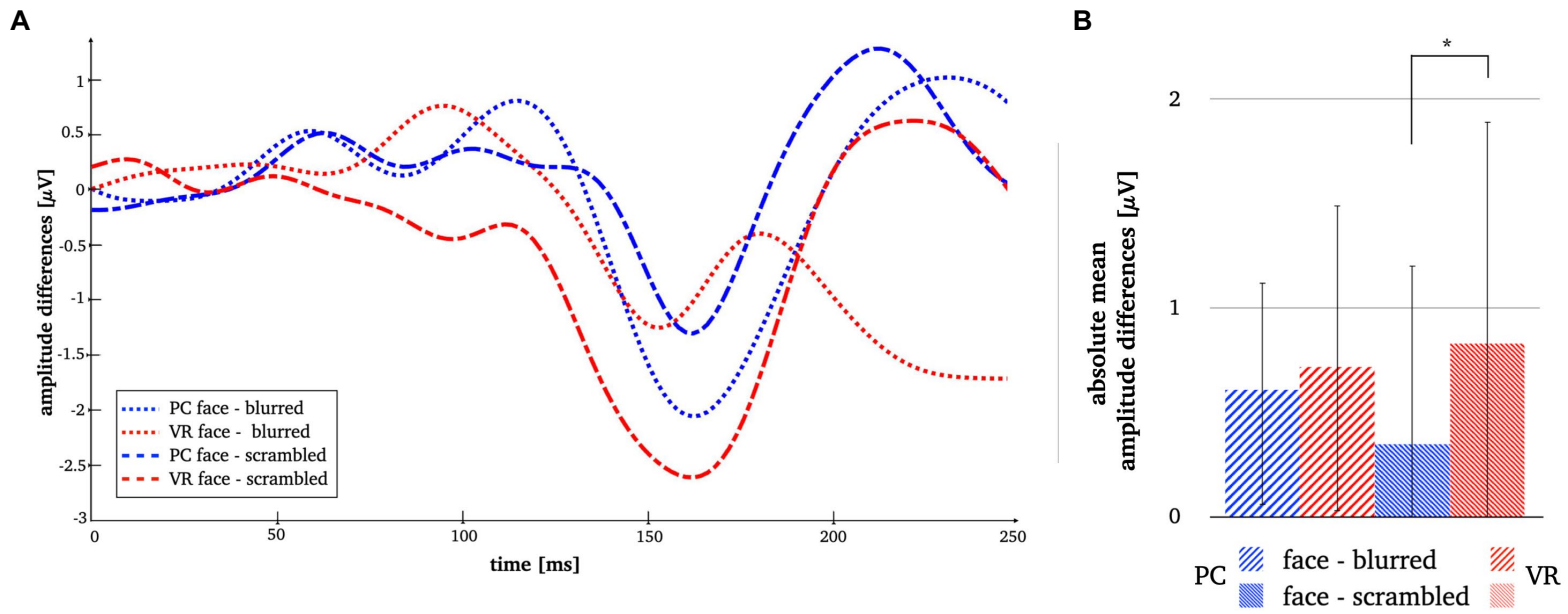
Panel **A** illustrates the N170 amplitude differences after subtraction of the perceptual controls in both modalities. Panel **B** depicts the absolute mean face amplitudes after subtraction of the perceptual controls in both modalities. The error bars depict the confidence intervals for the mean values. Significant differences are marked (*p < 0.05).

Comparing stimulus types across modalities revealed significantly more negative N170 amplitudes for all stimulus types in VR when compared to PC (see Table 3). Lastly, the analyses of interaction effects showed significantly more negative N170 amplitude for normal faces and blurred faces in VR when priorly subtracted by the scrambled type (see Table 3; Figure 9). For the results of the robust *t*-tests for all comparisons within and between modalities see Table 3.

#### 3.1.3. L1

**Figure 10:**
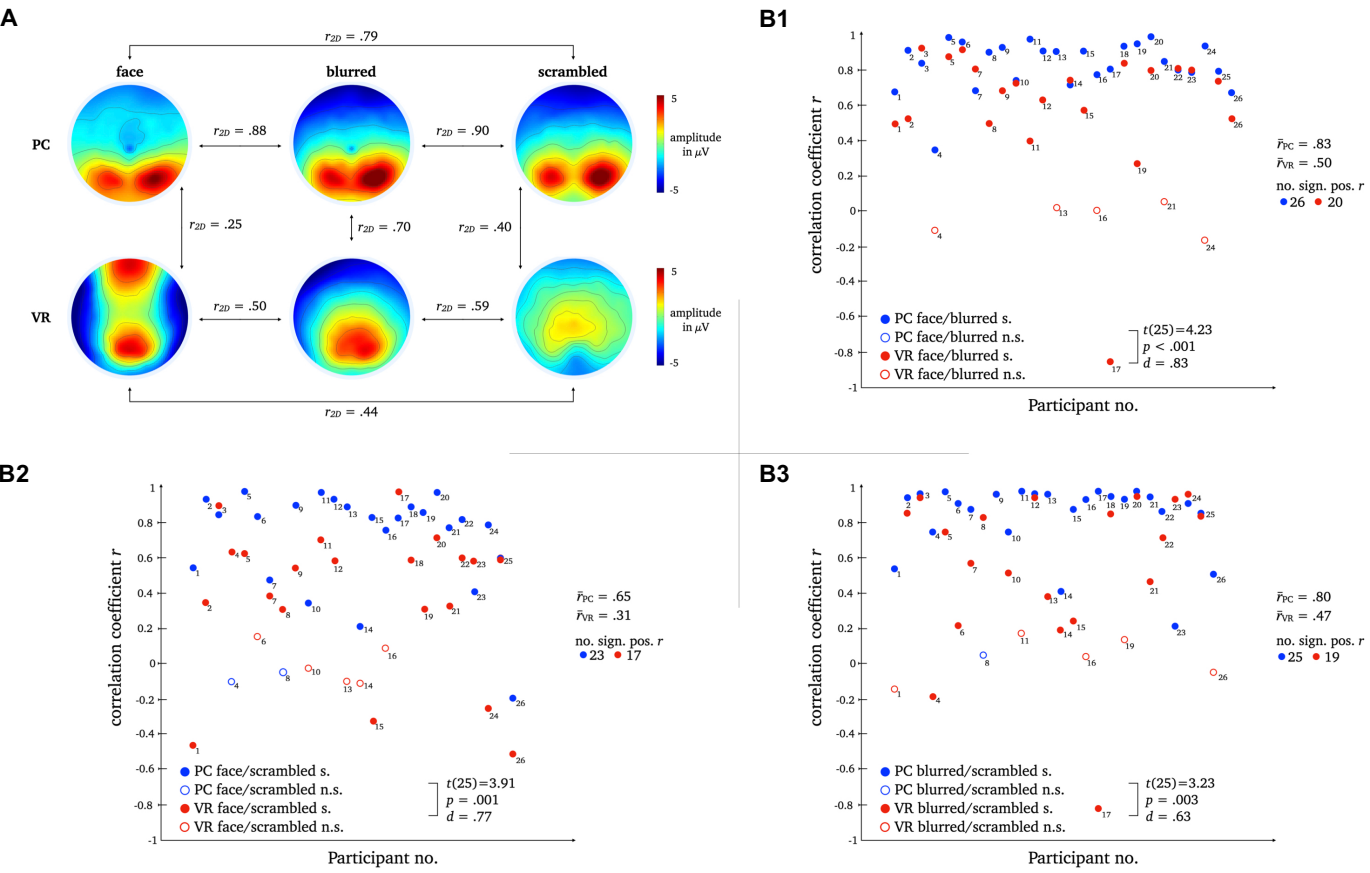
L1 topographies with 2D-correlation coefficient r for within and between modality comparisons [panel **A**]. The scatter plots illustrate individual correlation coefficients for stimulus type comparisons between modalities: Face vs. Blurred [panel **B1**], Face vs. Scrambled [panel **B2**] and Blurred vs. Scrambled [panel **B3**]. *T*-Test statistics, mean correlation coefficients and number of significant correlation coefficients are given.

A two-dimensional correlation as well as mean correlation coefficients for comparison between topographies within each modality revealed higher similarity between stimulus types within the PC condition. The results for the direct comparison of stimulus types across modalities also indicate low similarity. For detailed statistics please refer to Figure 10.

Pairwise *t*-tests of the correlation coefficients for stimulus comparisons between modalities showed strong differences between PC and VR. The similarity between the stimulus types is significantly lower within VR compared to PC, which is furthermore represented by a smaller number of significant correlations.

#### 3.1.4. L2

**Figure 11:**
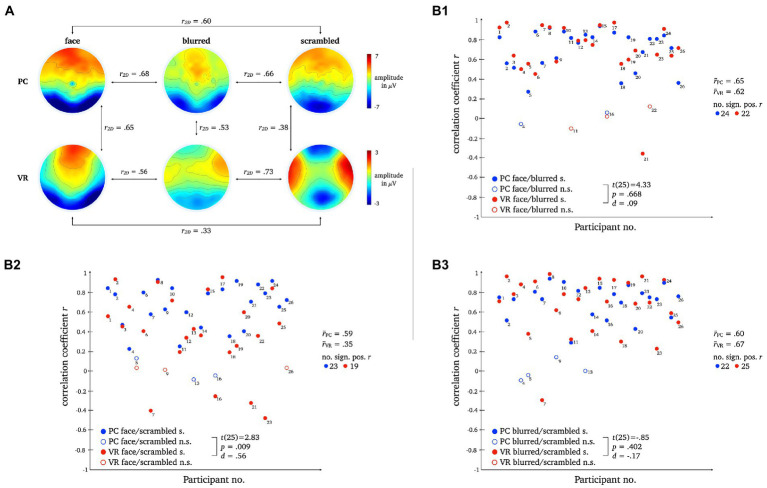
L2 topographies with 2D-correlation coefficient r for within and between modality comparisons [panel **A**]. The scatter plots illustrate individual correlation coefficients for stimulus type comparisons between modalities: Face vs. Blurred [panel **B1**], Face vs. Scrambled [panel **B2**] and Blurred vs. Scrambled [panel **B3**]. *T*-Test statistics, mean correlation coefficients and number of significant correlation coefficients are given.

The two-dimensional correlation as well as mean correlation coefficients for comparison between topographies within each modality disclosed bidirectional differences between stimulus types for PC and VR. The results for the direct comparison of stimulus types across modality indicate moderate similarity. For detailed statistics please refer to Figure 11.

Pairwise *t*-tests revealed moderate differences for normal compared to scrambled persons between VR and PC, but no significant differences for normal compared to blurred persons or blurred compared to scrambled persons were found.

### 3.2. ERP components in source space

#### 3.2.1. P1, N170 and L1 across modality and stimulus type

All components were localized to the bilateral occipital pole, with the center of gravity in the right hemisphere when averaged across conditions and stimulus types and were thus consistent with previous literature (e.g., Di Russo et al., 2002; Gruber et al., 2006; Figure 4).

*N170*: Regarding the N170 component, the difference between PC faces and VR faces was localized to the bilateral lingual gyrus with the center of gravity in the right hemisphere (see Figure 5A1). When controlling for blurred faces, the difference was localized to the bilateral lateral occipitotemporal gyrus (CG: left; see Figure 5B1), whereas it localized to the bilateral medial occipitotemporal gyrus (CG: left; see Figure 5C1) when controlling for scrambled faces.

*L1*: The difference between PC faces and VR faces yielded significantly different activity in the left inferior temporal gyrus regarding the L1 component (see Figure 5A2). When controlling for either blurred or scrambled faces, the differences in activity localized to the bilateral occipital pole (see Figures 5B2,C2). Interestingly, the difference between PC faces and VR faces was accompanied by significant differences in the activity of the cingulate region for the raw difference as well as when controlling for scrambled faces, but not when controlling for blurred faces (see Figure 6).

## 4. Discussion

The aim of this study was to investigate the neuronal mechanisms of realistic human face processing by translating the conventional laboratory setup into a realistic setting using VR. To take a first step towards bridging the gap between classical laboratory designs and reality, we followed the overall rationality of laboratory conventions, both in terms of experimental setup as well as the analytical methods, and thereby maintained comparability of the electrophysiological results.

To this end, randomized picture sequences of persons and two perceptual controls were presented to the participants in a blocked within-design (see Methods) under realistic conditions *via* a head-mounted display (VR modality) and under conventional 2D conditions *via* a PC monitor (PC modality). In our VR condition, participants were confronted with three-dimensional, real-life-sized persons sitting directly in front of them, undercutting social distance and invading the personal space. Due to the spatial proximity of the VR stimuli, a feeling of social involvement is created thus requiring processing of complex contextual and self-relevant information. To control for low-level perceptual differences (e.g., size and shape) between the two presentation modalities, affecting cognitive processing, the across modality comparisons were carried out after subtracting the amplitudes of the control images (i.e., blurred and scrambled pictures). This allows for comparing the N170 amplitudes between PC and realistic conditions beyond basal perceptual processes of the stimuli’s low-level visual features (e.g., size, shape, color). The control images thus served as a perceptual baseline. Albeit reporting results from robust statistical methods, in line with existing laboratory research, we base our interpretations on the conventional *t*-test.

By investigating classical ERP components commonly related to face processing, i.e., N170 (e.g., Itier and Taylor, 2004; Blau et al., 2007; Rossion and Jacques, 2011) and P1 (Itier and Taylor, 2002; Herrmann et al., 2005a; Thierry et al., 2007; Dering et al., 2009; Kuefner et al., 2010), and later components (L1, L2) as well as their cortical generators, we compared face processing mechanisms between conventional laboratory and realistic conditions by means of VR. Most importantly, our results under laboratory conditions generally replicate previous studies showing no indication of a face sensitivity of the P1 (e.g., Jemel et al., 2003; Ganis et al., 2012), a face specificity of the N170 when presented on a PC monitor (e.g., Eimer, 2000; Jemel et al., 2003; Rousselet et al., 2008; Rossion, 2014; Civile et al., 2018) and the relevance of later components in perceptual processing (e.g., Herbert et al., 2013; Bublatzky et al., 2014; Schindler et al., 2017). Although the ERPs obtained under laboratory conditions exhibited the same topographies at early stages of face processing, at later stages they differed markedly from their VR counterparts. Especially the L1 exhibited superior discrimination between faces and controls only in VR. These results indicate a more fine-tuned processing of faces under realistic conditions in VR, casting doubt on the general meaningfulness of the N170 as a singular marker for real-life face processing.

### 4.1. ERP components in electrode space

#### 4.1.1. P1

Investigations concerning a potential face sensitivity of the P1 component yielded no significant main effects, i.e., no discrimination between stimulus types in either modality.

The P1 seems to be essentially sensitive to certain stimulus properties but not specifically for faces, when presented on a PC monitor or a VR headset. In line with previous study results, the P1’s stimulus sensitivity presents a rather inconclusive picture. Whether the P1 is fundamentally insensitive for faces (see, e.g., Ganis et al., 2012), sensitive for object category (see, e.g., Thierry et al., 2007; Dering et al., 2009) or even task-sensitive (see, e.g., Dering et al., 2009) is unclear.

Under realistic conditions, the P1 did not discriminate between any stimulus types and thus showed no face sensitivity at all. These results extend previous research and suggest that the P1 is not a suitable neural correlate for the perception and processing of faces under realistic conditions.

#### 4.1.2. N170

Most importantly, confirming previous results on the N170 component face effect, we replicated other laboratory studies finding stronger amplitude deflections for 2D faces compared to non-face perceptual controls (e.g., Eimer, 2000; Jemel et al., 2003; Rousselet et al., 2008; Rossion, 2014). Furthermore, we extended results from other experimental setups using 3D-presentations that obtained the N170 using VR (Stolz et al., 2019) by comparing it directly to conventional laboratory conditions.

The comparison of the N170 amplitudes within each modality revealed no significant differences between faces and controls within the VR modality. Even though the *p*-values of the amplitude differences under realistic conditions reached trend level, no evidence for the discrimination of stimulus types by the N170 could be found. Thus, the N170 loses some of its discriminatory power when obtained under more realistic conditions with VR.

When contrasting the N170 amplitudes of the three stimulus types directly across modalities, VR leads to more negative amplitudes than PC. Considering the comparably larger stimulus size, the area occupied on the retina and the retinotopic organization of the visual cortices, respectively, these amplitude differences are most likely result for the stimuli’s physical perceptual features (Busch et al., 2004; De Cesarei and Codispoti, 2006; Josef Golubic et al., 2011; Pfabigan et al., 2015). Previous research has shown that the perception of the human body independent from the face, also elicits a typical N170 response, in some cases with a delayed latency, i.e., a N190 response (Stekelenburg and De Gelder, 2004; Thierry et al., 2006). In both conditions of this study, we deliberately used stimuli that comprised the face as well as upper body of persons as one would encounter it in real life as well. The amplitudes are therefore influenced by the perception of the upper body as well, but this affects both conditions which is why it is unlikely to explain the amplitude differences between the modalities.

The interaction effects across modality, i.e., subtracting the amplitudes of the control pictures before comparing the N170 amplitude, revealed stronger N170 deflection under realistic conditions for one of the perceptual baselines. Controlling for perceptual frame and color information by subtracting the amplitude of the blurred image, led to comparable N170 amplitudes in both modalities. The subtraction of the amplitude of the scrambled image, i.e., low-level perceptual visual features, resulted in a stronger N170 deflection for VR faces. However, the amplitude difference face *minus* scrambled was not significantly different from zero, providing further evidence against the specificity for faces under realistic conditions. Hence, the modality effect actually results from smaller variances within the 2D modality, showing that the N170 face specificity can be replicated in the conventional laboratory but not under realistic conditions.

Taken together, differences between the N170 obtained under realistic conditions as opposed to conventional laboratory conditions are apparent but relatively small, which is reflected by equally small effect sizes (see Results). Our results confirm the long hold notion that the N170 specifically reflects cognitive processes related to face perception under conventional 2D PC conditions, while our study does not provide any evidence that under realistic conditions the N170 likewise indicates face perception as it does not differentiate between different types of stimuli. Thus, our data shows that the N170 specificity seems to be a domain or modality related effect (Schöne, 2022). The comparison of perceptual-baseline-corrected amplitudes furthermore showed that the amplitude variations between stimulus types are rooted in the PC effects. It has yet to be determined what might be the crucial factor diminishing the meaningfulness of N170 under realistic conditions. So far, one can only speculate as this study only took a first step in that direction. However, as described in the introduction, considering the much more immersive character of a visual scene in VR in which a presented person is embedded and the spatial proximity with which it can be done, it seems likely that the encounter with a human face under such conditions – as it would occur in real life – requires complex cognitive processing that differs from what we find when people watch faces on computer screens.

Previous studies have shown that the face specificity of the N170 cannot be replicated under all conditions, even in the conventional laboratory. For example, the N170 amplitude has been shown to also be sensitive to factors such as inter-stimulus perceptual variance (ISPV; Thierry et al., 2007; Dering et al., 2009). In these studies, ISPV significantly modulates the N170 amplitude while the object category (i.e., face or car) does not. Moreover, cars produced stronger N170 deflections than faces (Dering et al., 2009), raising doubt regarding the N170 face effect. Results on the inversion effect on face perception obtained in a discrimination task using faces and cars show that the sensitivity of the N170 could also be explained by topographical differences and stimulus specific neural generators (Boehm et al., 2011). Most importantly, the N170 does not reflect behavioral improvements in social functions (Key and Corbett, 2020), which further questions whether the N170 reliably indexes face perception to be generally applicable even outside the conventional laboratory. Reflected in the results of this study, the N170’s face specificity is only obtainable for faces that were presented on a PC monitor, but not consistent when transferred to realistic conditions.

#### 4.1.3. L1 and L2

In contrast to the results of the N170 component, the investigation of the late components revealed the opposite picture. The correlation analyses yielded higher similarity between topographies of the three stimulus types within PC as opposed to VR. The PC topographies are very similar suggesting that the same object perception mechanism is used for faces and controls, i.e., very different stimulus types. In contrast, the VR topographies differ considerably, implying distinct neural mechanisms for perception of faces, silhouettes and objects. Moreover, the topographies for each stimulus type differ between PC and VR, further supporting the stimulus specificity of the neural mechanisms applied under realistic conditions. The L1 component clearly differentiates more effectively between stimulus types under realistic conditions.

The results for L2 tie in well with the results for L1. The PC topographies are very similar as well, while the VR topographies are much more distinct. Across modalities, the topographies for faces and blurred controls look moderately similar, but for scrambled controls still markedly different. Again, the increased differentiation between face and object perception due to greater topographic differences under realistic conditions suggests face-specific neural mechanisms operating when encountering a realistic face that are not required for the monitor. Hence, as initially considered, examining face perception as an isolated process using typical face stimuli that are outside an egocentric reference frame and devoid of social context, initiates domain specific neural mechanisms that do not possess the same functional properties as those required for real-life face processing.

Extending previous laboratory studies, later components reflect said mechanisms of realistic face processing. In contrast to earlier components, later potentials are linearly related to stimulus realism (Schindler et al., 2017), modulated by socially relevant emotional expressions and affective contexts (Bublatzky et al., 2014; Stolz et al., 2019) and especially sensitive for self-related emotions (Herbert et al., 2013). Processing of actually self-relevant emotional and contextual information, such as, e.g., threat towards oneself, seems to not be captured by the N170 component. Thus, consistent with laboratory results, late components discriminate faces and controls under realistic conditions, as they exhibiting much more discriminatory potential than the N170.

### 4.2. ERP components in source space

#### 4.2.1. N170

The source analysis of the N170 resulted in modality differences in the lateral and medial occipitotemporal gyrus revealing differing activation under VR and PC conditions. The medial occipitotemporal gyrus, which comprises the lingual gyrus, the parahippocampal gyrus, and the lateral occipitotemporal gyrus, which is also known as the fusiform gyrus, are functionally connected and involved in higher-order visual processing (Mccarthy et al., 1997; Weiner and Zilles, 2016; Palejwala et al., 2021; Williams, 2021). Especially the fusiform gyrus is specialized in face perception and object recognition (Mccarthy et al., 1997; Weiner and Zilles, 2016). The lingual gyrus, linking fusiform and parahippocampal gyrus, is related to processing of complex visual stimuli and their basic characteristics, such as emotional facial expressions, and moreover provides access to visual memory storage (Kozlovskiy et al., 2014; Palejwala et al., 2021). The parahippocampal gyrus is associated with a neural network processing contextual associations (Aminoff et al., 2013) and related to assessment of spatial configurations of objects while not determining object identity (Bohbot et al., 2015). With regard to the results at hand, the obtained neural generators of the N170 suggest a first basic processing of faces on a primarily sensory-perceptual level that allows recognition of the stimulus being a face under both, laboratory and realistic conditions. However, taking into account the ERP results, a N170 face specificity is evident only under laboratory conditions, whereas under realistic conditions, only a low-level sensitivity is observed.

#### 4.2.2. L1

For the source analysis of the L1 component, modality differences emerged in the left inferior temporal gyrus and cingulate region. The inferior temporal gyrus constitutes a higher level of visual processing, merging various higher cognitive functions such as visual perception, emotion regulation and memory (Miyashita, 1993) and is moreover related to person-specific semantic knowledge (Giovanello et al., 2003) as well as face individuation (Nestor et al., 2008; Pyles et al., 2013). As a core midline structure, the cingulate region is part of a network responsible for self-referential information processing (Northoff et al., 2006) and due to its functional connection to the hippocampus and the amygdala, it forms an important connection hub playing a role in long-term memory processing of emotional relevance of stimuli (Bubb et al., 2017; Rolls, 2019), such as familiar faces (Pierce et al., 2004). In contrast to the N170, the neural generators of the L1 reveal involvement of complex face-specific cognitive functions, such as memory and emotion regulation, suggesting more in-depth processing of the presented faces, i.e., consulting self-referential information and recognition of familiar faces. These assumptions are in line with aforementioned ERP findings in face perception, showing late components to be modulated by contextual, emotional and self-related information.

Taken together, contrasting neural sources under conventional as opposed to realistic conditions revealed an overall picture of face perception that would be expected when investigating realistic, self-relevant face processing. Initially, still within the time course of the N170, a face is detected as such, including recognition of its spatial configuration and contextual associations which is further supported by access to visual memory storage. However, processing of emotional relevance and retrieval of self-relevant face-specific information transpires on a broader time scale, reflected by the L1 component. Here, a face is recognized as an individual object with an identity and actual relevance within the observer’s self-reference frame. Thus, the evaluation of emotional value and the automatic search for familiarity of an encountered face are specific to realistic conditions and are not reflected by results from the conventional laboratory.

## 5. Conclusion

The translation of conventional laboratory conditions into a novel, more realistic setup in VR presents a first step towards the investigation of real-life face perception. To our knowledge, this is the first study directly comparing face perception between a conventional 2D monitor setup and realistic conditions using VR.

In line with previous laboratory studies, our ERP analysis confirms that the N170 does seem to be face-specific, however, only to a certain degree as it loses considerable discriminatory power in VR. These results raise doubt to the N170 as a meaningful marker for real-life face processing and our study implies it to be domain-specific, i.e., specific to the monitor. Specifically, its discriminatory capability is only applicable to planar, two-dimensional and unresponsive, but not real-life faces.

Our results on later components reveal distinct mechanisms for faces, silhouettes and objects being applied under VR conditions as opposed PC conditions. This is further supported by an in-depth source analysis suggesting a tripartite processing structure: First, early detection of perceptual face characteristics, second, registration of emotional value, and finally self-relevant retrieval of, and comparison with familiar faces of which any processing beyond basic perceptual properties is manifested in later components.

Hence, our study is in line with previous studies contrasting electrophysiological markers obtained under 2D with VR conditions, providing evidence that said markers and the functional neural properties they reflect are specific to the domain in which they occur. In a study on frontal-alpha-asymmetry (FAA) by Schöne et al. (2021a,b) it became apparent that the FAA does not index the same emotional and motivational state in both modalities, 2D and VR. Likewise, in a memory paradigm, the theta old-new-effect could not be replicated when the stimuli were first presented in VR (Kisker et al., 2021b). On a more general note, researchers should be aware that their findings do not necessarily translate to realistic conditions and should therefore be careful when generalizing their results beyond the setting they were observed (Yarkoni, 2022). Specifically, cognitive and emotional processes might not generalize beyond the conditions under which they are measured (see also Schöne, 2022).

To further investigate why and how the N170’s face specificity seems to not be consistent when obtained under realistic conditions in VR, more research in this direction is needed. It would be of great interest to investigate face processing in VR further by comparing the perception of faces to other object categories (e.g., cars) and to give participants the task to actively discriminate faces from controls (e.g., button press). Taken from our results, it seems to be promising to take a look beyond the timeframe of the N170 – potentially towards later components such as the L1 - to find a real-life neural marker for face processing.

In summary, face perception is a complex interplay of neural mechanisms occurring on a broader timeframe than roughly 200 ms post stimulus presentation. The present study confirms conventional laboratory results, which provide first evidence for the relevance of late ERP components in face processing, and further extends this assumption to realistic conditions. By means of correlation analysis and identification of neural generators, we showed that realistic face perception includes early face detection, in parts captured by the N170. Beyond basic-level processing, however, face perception seems to require emotion assessment as well as self-relevant retrieval of and comparison with familiar faces, only reflected in late components that are only captured under realistic conditions.

### 5.1. Limitations

Even though VR as a research tool offers the possibility to increase realism under laboratory conditions, the experimental design used in the present study is nevertheless modeled after conventional laboratory setups. It is not our ambition to introduce a parallel research discipline employing VR, but to stay in line with previous research results on face perception, and to gradually bring the conventional laboratory closer to reality. The sequential presentation of numerous static, unresponsive stimuli in a block design that are appearing suddenly in front of the participant is still physically implausible and does not correspond to a real-life scenario. Most importantly, however, it allows for comparison of ERPs between modalities. The implementation of dynamic faces within a meaningful context and moreover, the possibility to respond to them can further increase the realism of the experimental setup. Thereby, an even closer approximation to real-life face processing will be achieved. The present results should be extended by frequency analysis in addition to ERP analysis, which is a promising cognitive manifestation involved in face perception and characterization (Zion-Golumbic and Bentin, 2007). Moreover, inquiring subjective measures of participants, e.g., arousal, valence and presence, would give further insight into the participant’s perception of the VR modality.

It should be mentioned at this point that a great deal of studies on the N170 focus on the comparison of faces with inverted controls or other morphological modifications that are not easily translatable to VR, simply because they would appear extremely irritating to the participant. Consequently, the comparability to studies using these kinds of controls is limited. However, it could be considered to implement a similar design in VR in future studies to investigate whether the same effects are obtainable under realistic conditions and to consider later components here as well.

## Data availability statement

The datasets presented in this study can be found in online repositories. The names of the repository/repositories and accession number(s) can be found at: https://osf.io/y8c6q/?view_only=0d2fd8d6bd1e4351afe0deb5e3f4d3a4.

## Ethics statement

The studies involving human participants were reviewed and approved by the local ethic committee of Osnabrueck University, Germany. The patients/participants provided their written informed consent to participate in this study. Written informed consent was obtained from the individual(s) for the publication of any identifiable images or data included in this article.

## Author contributions

All authors contributed to the study concept and design. JK and MJ developed the Unity VR environment, while BS provided the 2D stimulus presentation program. Testing and data collection was performed by MS and JK. Main data analyses, interpretation and visualization were performed by MS under supervision of BS and TG. Source analysis was performed and respective results were drafted by JK. Additional data anylsis using robust statistics was performed by SS, respective parts of the manuscript were also drafted by SS. MS drafted the manuscript, MJ and JK revised the manuscript. BS and TG provided critical revisions. All authors approved the final version of the manuscript for submission.

## Funding

We acknowledge support by Deutsche Forschungsgemeinschaft (DFG) and Open Access Publishing Fund of Osnabrück University.

## Conflict of interest

The authors declare that the research was conducted in the absence of any commercial or financial relationships that could be construed as a potential conflict of interest.

## Publisher’s note

All claims expressed in this article are solely those of the authors and do not necessarily represent those of their affiliated organizations, or those of the publisher, the editors and the reviewers. Any product that may be evaluated in this article, or claim that may be made by its manufacturer, is not guaranteed or endorsed by the publisher.

## Footnotes

^2^https://github.com/sccn/labstreaminglayer
